# Assessing the use of the neighborhood method to estimate the prevalence of child separation: a pilot in North Kivu, DRC

**DOI:** 10.1186/s13031-016-0084-7

**Published:** 2016-08-17

**Authors:** Hani Mansourian, Beth L. Rubenstein, Craig Spencer, Makini Chisolm-Straker, Eva Noble, Anna Skeels, Chiara Ceriotti, Lindsay Stark

**Affiliations:** 1 Program on Forced Migration and Health, Mailman School of Public Health, Columbia University, 60 Haven Avenue, New York, NY 10032 USA; 2 Department of Epidemiology, Mailman School of Public Health, Columbia University, New York, USA; 3 Department of Emergency Medicine, The Icahn School of Medicine at Mount Sinai, New York, USA; 4 Humanitarian Department, Save the Children UK, London, UK

**Keywords:** Democratic Republic of the Congo, Child protection, Unaccompanied and separated children, Prevalence, Neighborhood method, Household survey

## Abstract

**Background:**

This article reports on the use of the ‘neighborhood method’ to measure the prevalence and basic characteristics of children who became separated from their parents or usual caregivers subsequent to an attack by the M23 militia group in North Kivu, Democratic Republic of the Congo.

**Methods:**

A two-stage household cluster survey was conducted in 522 households in North Kivu in August 2014. Heads of households were asked about separated children in their household, as well as the households of their two closest neighbors. Separation was tracked in terms of children who arrived into the households after the M23 attacks and children who departed from the households after the recall event without their parent(s) or usual caregiver. For a subset of 44 neighbor pairs, respondents were asked to report on the same household to assess inter-rater reliability. Data about primary respondents and their neighbors were assessed to determine whether the neighborhood method was a comparable, reliable and efficient alternative to a traditional household survey about separated children.

**Results:**

The prevalence of separated children who arrived was 8.52 % [95 % CI: 6.75–10.75] in primary households and 4.46 % [95 % CI: 3.60–5.52] in neighbors’ households (*p*-value = 0.0000). The prevalence of separated children who departed was 4.98 % [95 % CI: 3.45–7.19] in primary households and 3.19 % [95 % CI: 2.27–4.48] in neighbors’ households (*p*-value = 0.0110). Kappa coefficients for the neighbor pairs indicated fair to moderate agreement for most demographic variables, but agreement was generally higher for variables related to current characteristics of the households than for variables describing the household in the past, especially before the M23 attack. Compared to a traditional household survey with similar power, the neighborhood method reduced data collection time by 50 % and lowered costs by 36 %.

**Conclusion:**

This pilot showed that, for measuring separated children in North Kivu, the results from neighbor households significantly underestimated the prevalence of separation when compared to data collected from respondents directly. Reliability was mixed. Although the neighborhood method did not yield valid results in this setting, given the potential the method holds to save scarce resources in humanitarian settings, additional pilots to refine and evaluate its validity and reliability in settings with shorter recall periods are recommended.

## Background

It is well documented that children who are separated from their parents or usual caregivers face a multitude of risks [1, 2]. Compared to children who are not separated, these children have an increased likelihood of recruitment and abduction into armed forces and groups [3]. They suffer from higher levels of food insecurity, and are more likely to be exploited for labor and sex than their unseparated peers [4–6]. In addition, separation can have long-term social and psychological impacts, including chronic stress and anxiety [7, 8].

Recognizing these risks, programs to address the needs of separated children have become a cornerstone of child protection in emergency response, dating back to shortly after World War II [9]. However, while minimum standards exist to guide organizations in establishing family tracing, reunification and alternative care programming, there are currently no guidelines for quantifying the overall magnitude of such separation in an emergency [10]. As a result, practitioners and policymakers are left to assess the scope of separation, for programming purposes, based on gross generalizations and/or selective data. The most common strategy to assess magnitude is to employ a “rule of thumb” which suggests that unaccompanied and separated children (UASC) typically comprise 3–5 % of the displaced population during emergencies [11]. This approach has never been validated. Other mechanisms rely primarily upon key informants employing a “best guess” at the scale of separation in their community subsequent to the emergency of interest.

There is thus a pressing need for reliable, valid and feasible population-based methods to estimate the prevalence of separated children in emergencies. Population-based prevalence data, such as that generated from a household survey, has enormous potential to inform funding, programming, and policies for separated children. However, due to security and accessibility constraints and limited time, finances and human resources, it is difficult to use conventional household surveys to estimate the prevalence of children who are separated from their usual caregivers in emergencies. These challenges are exacerbated by the fact that separation is generally a relatively rare and hidden event and thus requires a large sample size to achieve adequate statistical power.

The neighborhood method was developed in an attempt to overcome some of the logistical challenges associated with conducting a household survey in a complex emergency [12]. The neighborhood method is an adapted household survey approach whereby randomly sampled households are asked to provide information about their household, as well as the households of their neighbors. The neighborhood method has proven useful for measuring sensitive events such as sexual violence in situations where security, logistical, and financial limitations make large samples difficult to attain [12–14].

Working in conjunction with the global Child Protection Working Group, a survey tool was developed and piloted by Columbia University researchers to measure the prevalence and basic characteristics of unaccompanied and separated children in a defined area, affected by the same emergency.^1^ The tool asked respondents to provide information about separated children from their own household, as well as separated children from the households of their two closest neighbors. Here, the investigators evaluate the findings about primary respondents and their neighbors to determine whether the neighborhood method was a comparable, reliable and efficient alternative to a traditional household survey about separated children.

## Methods

### Setting

The neighborhood method was tested as part of a broader pilot project that aimed to measure the prevalence and basic characteristics of separated children in emergency contexts [15]. The pilot project was implemented between July and August 2014 in Nyiragongo territory and the town of Goma, two areas in the North Kivu province of Democratic Republic of the Congo (DRC). These areas have been affected by armed conflict for more than two decades [16]. Children in the region are regularly separated from their families due to violence, displacement, poverty and recruitment to armed forces [17, 18]. In late 2012, a militia group known as M23 attacked these areas, overtaking the city of Goma and surrounding areas, displacing parts of the population and exacerbating the conditions that lead to separation.

### Sample

Sampling was achieved via a two-stage cluster design. The sample was powered for a traditional household survey (i.e. not considering the increase in sample size due to use of the neighborhood method). Twenty clusters of 25 households per cluster were targeted in order to detect a 5 % prevalence of separation in a population of 10,000, assuming precision of 1.5 % and a design effect of two. Due to insecurity in many parts of the covered areas, clusters were randomly selected from areas identified as accessible by the security team at the hosting organization. To select households within each cluster, systematic random sampling was used. Primary and neighbor households were identified using a fixed interval calculated based on the estimated number of households in the sample area. Households that were a multiple of the interval were the primary households and the two next most proximate households (as determined by the survey team leader) were the neighbor households. If two houses were equally close to the primary household, a coin flip was used to randomly select amongst the two. The survey team leader was responsible for preventing duplication by ensuring that no household was included as both a primary and a neighbor household during data collection. In each household, if no one over the age of 17 years was home, the next available house in the pattern was approached.

**Fig. 1:**
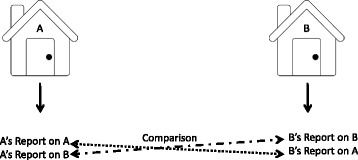
Schematic for reliability subsample

Adult respondents from 522 primary households were surveyed and asked to provide information about their own household and the households of their two closest neighbors. This resulted in an effective total sample size of 1533 households (522 primary households, 515 first neighbors, and 496 s neighbors). A sub-sample of 44 neighbor pairs was also selected to more directly assess the reliability of respondents’ reports about their neighbors. Each adult informant in the sub-sample of neighbor pairs was asked to report about his/her own home and the home of an adjacent neighbor, such that in a pair of adjacent dwellings, two separate reports about UASC for each home were obtained: one from the household of interest, and one from the neighbor [See Fig. 1].

### Measures

The survey intended to measure the prevalence and basic characteristics of children who were separated from their parents or usual caregivers in the aftermath of the M23 attacks of December 2012. As per the inter-agency guiding principles for unaccompanied and separated children, separated children were defined as children who have been separated from both parents, or from their previous legal or customary primary caregiver, though not necessarily from other relatives. Unaccompanied children were defined as children who have been separated from both parents and other relatives and are not being cared for by any adult who, by law or custom, is responsible for doing so [2].

Because separated and unaccompanied children may be living outside of a household (e.g. in a residential care facility, on the street, with an armed group), a household survey will inherently miss a segment of the population of interest. This was partially addressed in this study by capturing two distinct populations of children. First, we measured separated children who arrived, defined as separated or unaccompanied children who started living in the sampled household at any point after December 2012 (the date of the M23 takeover of Goma). Second, we measured separated children who departed, defined as children who left the sampled household after December 2012 and were separated from their usual caregiver. Children who departed included children living outside of the sampling universe. Births and deaths were not counted as children who arrived or departed.

### Study protocol

Heads of households were chosen as the primary respondents. Verbal consent was obtained from all participants. Questions were designed to ask about household composition in general before and after the emergency event of interest, rather than separated children in particular. This approach was intended to reduce bias in case respondents had an interest in either over- or under-estimating the true number of UASC.

As part of the interview, interviewers used cards to visually depict each household member. Cards were then arranged to ‘map’ the current household composition in comparison to the household composition before the M23 attacks. The interviewer asked the sex and age of each household member, his/her relationship to the head of the household and whether s/he was still alive. Where children who had arrived or departed since the emergency were identified, the interviewer asked additional questions about that child, including reasons for separation and current caregiver.

This study was covered under Columbia University Medical Center’s IRB reference AAAB7134.

### Data analysis

Data was analyzed using SAS 9.4 and Microsoft Excel. Standard errors were adjusted for clustering at the neighbor level by incorporating a Poisson regression model using the method of generalized estimating equations (GEE). Two sample t-tests were performed to evaluate the null hypothesis that mean household characteristics and levels of separation were equal across primary households and neighbor households. Kappa statistics were used to evaluate inter-rater reliability amongst paired neighbors. Feasibility was assessed according to four criteria: ease of training, interview time, data collection costs and social acceptability.

## Results

### Comparability of main outcomes

Five hundred and twenty-two primary households were surveyed. Of these 522 households, 98.7 % (*n* = 515) of respondents provided information about their first neighbors and 95.0 % (*n* = 496) provided information about their second neighbors, yielding a neighbor sample of 1011 households.

The prevalence of separated children who arrived was 8.52 % [95 % CI: 6.75–10.75] in primary households, meaning that in the sample of all 2197 children living in the respondents’ homes at the time of data collection, 186 were separated children who had arrived in the household since the M23 attack in December 2012. For separated children who departed, the prevalence in primary households was 4.98 % [95 % CI: 3.45–7.19], meaning that in the sample of all 2034 children living in the respondents’ homes prior to the M23 attack, 108 children had departed from the household, resulting in separation from their parents or usual caregivers. In neighbors’ households, the overall prevalence of separated children who arrived was 4.46 % [95 % CI: 3.60–5.52] and the overall prevalence of separated children who departed was 3.19 % [95 % CI: 2.27–4.48]. Both of these differences in overall prevalence rates between primary households and neighbors’ households were statistically significant at the 5 % level (see Table 1).

**Table 1:** Prevalence of separation by primary households and neighbors’ households

	Primary households			Neighbors’ households			
	*n*	Prevalence	95 % CI	*n*	Prevalence	95 % CI	*p*-value
Arrivals							
Separation (overall)	186	8.52 %	(6.75–10.75)	154	4.46 %	(3.60–5.52)	0.0000
in villages	164	9.07 %	(7.11–11.56)	112	4.14 %	(3.19–5.37)	0.0000
in camps	22	5.88 %	(3.08–11.24)	42	5.64 %	(4.92–6.47)	0.8830
Unaccompaniment	41	1.81 %	(1.19–2.75)	65	1.78 %	(1.17–2.69)	0.8520
Departures							
Separation (overall)	108	4.98 %	(3.45–7.19)	99	3.19 %	(2.27–4.48)	0.0110
in villages	60	3.37 %	(2.37–4.80)	66	2.60 %	(1.87–3.62)	0.2120
in camps	48	11.29 %	(9.21–13.83)	33	5.85 %	(3.20–10.67)	0.0380
Unaccompaniment	11	0.44 %	(0.20–0.97)	6	0.19 %	(0.08–0.50)	0.1800

The trend of primary respondents reporting significantly lower separation prevalence in neighbors’ households, compared to their own households, was seen in several more specific measures as well, most notably among separated children who arrived in villages and separated children who departed in camps. A similar trend of lower separation prevalence in neighbors’ households was detected in all other categories analyzed (separated children who arrived in camps, separated children who departed from villages and unaccompanied children who arrived and departed across all locations), but these differences were not statistically significant. When prevalence rates were further disaggregated by first reported neighbor households and second reported neighbor households, there was also a slight trend towards lower reported prevalence of separation in second neighbor households, compared to first neighbor households. However, again, these differences were not statistically significant.

To better understand what may have driven these differences in reported prevalence rates between primary households and neighbors households, additional demographic variables were compared across both groups. Specifically, there was a trend for primary households to report approximately one less person in their neighbors’ households compared to their own households. This statistically significant trend was consistent for total numbers of people per household, as well as number of children per household (see Table 2).

**Table 2:** Household size by primary households and neighbors’ households

	Primary households (*n* = 522)		Neighbors’ households (*n* = 1011)		
	Mean	95 % CI	Mean	95 % CI	*p*-value
Current household size	6.43	(5.95–6.95)	5.43	(4.70–6.27)	<0.0001
in villages	6.70	(6.46–6.95)	5.52	(4.80–6.36)	<0.0001
in camps	5.38	(4.25–6.81)	4.60	(3.80–5.56)	0.0010
Current number of children	4.21	(3.87–4.58)	3.31	(2.08–5.27)	<0.0001
in villages	4.37	(4.17–4.57)^a^	3.41	(2.83–4.11)	<0.0001
in camps	3.59	(3.16–4.03)	2.98	(2.32–3.83)	<0.0001

^a^The Poisson regression using the GEE method to adjust for clustering between neighbors did not converge

### Reliability

Using the Landis and Koch interpretation, kappa coefficients indicated fair to moderate agreement for most variables for the 44 neighbor pairs reporting on the same household [19]. Agreement was highest for number of newborns (Kappa = 0.542, 95 % CI: 0.307–0.776)^2^, number of arrivals (Kappa = 0.445, 95 % CI: 0.081–0.809) and current number of children in the household (Kappa = 0.409, 95 % CI: 0.240–0.578). Agreement was lowest for all variables describing the household before the M23 attack, including the number of children living in the household before the emergency (Kappa = 0.181, 95 % CI: 0.010–0.352) and number of departures (Kappa = 0.189, 95 % CI: −0.233–0.612). All results had wide confidence intervals due to the small sample size (see Table 3).

**Table 3:** Agreement between neighbors

Variable	N	Kappa (unweighted)	95 % CI	*p*-value (2-sided)
Current household size	44	0.357	(0.184–0.530)	<0.0001
Current number of children	44	0.409	(0.240–0.578)	<0.0001
Number of newborns	44	0.542	(0.307–0.776)	0.0001
Number of arrivals	42	0.445	(0.081–0.809)	<0.0001
Household size before emergency	37	0.233	(0.052–0.415)	<0.0001
Number of children before emergency	40	0.181	(0.010–0.352)	0.0099
Number of departures	40	0.189	(−0.233–0.612)	0.1236

### Feasibility

#### Ease of training

The survey components related to the neighborhood method repeated the same questions and probes used to interview the primary households. Therefore, once data collectors understood the core questionnaire and interview guide, introduction of the neighborhood component was relatively straightforward. Out of the seven-day training course for data collectors, less than one day was devoted to training on the neighborhood method. Most of this time was spent ensuring the data collectors understood the protocol for choosing the closest neighbors based on geographic proximity before entering the selected primary household. Because this concept was both important and difficult, during implementation, members of the training team led the data collectors in identifying the closest neighbors for each primary household.

#### Time

Each interview required an average of 45 min to complete, including the neighborhood component. On average, approximately 15 min of each interview was dedicated to introduction and informed consent, 15 min to the primary household questionnaire and 15 min to the first and second neighbors’ questionnaires. That is to say, the neighborhood component of the survey only represented about a third of the total interview time. Thus, based on the observed attrition rate of 5 % for reporting on the second neighbor in this study, we estimated that using the neighborhood method would have enabled us to interview just 183 primary households to achieve a sample size of 522 households. Accordingly, if the survey had included neighbors’ data as part of the main sample, the total data collection time could have been halved, from 24 days to 12 days.

#### Cost

The estimated cost of collecting data from 183 primary households, including asking about their two neighbors (i.e., the neighborhood method), was compared to the cost of sampling 522 primary households, but not asking about neighbors (i.e., a traditional household survey). The cost of data collection with the neighborhood method would have been 36 % lower than the cost of a traditional household survey, or $12,059 versus $18,951, respectively.

#### Social acceptability

The vast majority of respondents agreed to provide information on both their first and second neighbors (98.7 % and 95.0 %, respectively). Anecdotal observations from data collectors also confirmed that respondents were willing to provide information about their neighbors’ household composition, including information about children who arrived and departed.

## Discussion

The purpose of this investigation was to determine whether the neighborhood method was a comparable, reliable and efficient alternative to a traditional household survey about UASC. From an implementation perspective, the neighborhood method proved to be a feasible methodology for measuring the prevalence of UASC in an emergency context. The method was simple to learn, significantly more time-efficient and cost-effective than a traditional household survey, and socially acceptable.

From a methodological perspective, suitability of the neighborhood method as a substitute for a traditional household survey depends on the strength of its underlying assumptions. The neighborhood method assumes that respondents’ neighbors are essentially random and representative of the general population; that respondents are aware of the current and past composition and care status of children in their neighbors’ households; and that respondents do not have reasons to over- or under-report the numbers of people or unaccompanied and separated children in their own or their neighbor’s household [14]. If these assumptions are correct, one would expect the findings from primary households to be similar to the findings from neighbors’ households. Instead, our data showed statistically significant differences for several key measures compared.

These differences between primary households and neighbor households could be driven by multiple factors. First, primary respondents may have deliberately underreported the number of children who arrived and departed in their neighbors’ households. The most plausible explanation for deliberate underreporting of the number of children who arrived and departed in neighbors’ households is respondent fatigue. Respondents may have realized during the first part of the interview about their own household that every arrival and departure identified triggered a new set of questions pertaining to the circumstances surrounding the child’s separation [20]. This may have led respondents to deliberately avoid reporting arrivals and departures to facilitate swift completion of the interview. The trend towards lower prevalence of separation in second neighbors’ households, compared to first neighbors’ households, is consistent with this theory.

A second possible explanation for the differences between primary respondents and neighbor households is that primary respondents did not have full knowledge of the composition of their neighbors’ households or of the presence of children who arrived in or departed from their neighbors’ households. The limited agreement between neighbor pairs reporting on the same household, especially with regards to historical data, suggests that incomplete knowledge of neighbors’ household composition may be a factor in the observed differences. This would also invalidate a central assumption of the neighborhood method that people know about the households of their neighbors. This theory could be tested in future applications of the neighborhood method by alternating the order of the questionnaire, such that some respondents first report on their own household (followed by their neighbors) and other households first report on their neighbors (followed by themselves).

A third conceivable explanation for the differences between primary respondents and neighbor households is that, due to the method in which households were sampled, neighbors’ households might be truly smaller than primary households on average. This bias could have arisen because a condition for conducting an interview with a primary household was the presence of an adult at the time the household was visited. The probability of an adult being home at the time of study visit likely increases with the total number of adults living in the household. Compared to larger households, smaller households were thus more likely to be excluded from the primary household sample, but not from the sample of neighbors’ households. However, true differences in size between the sample of primary households and the sample of neighbors’ households does not explain the low kappas between neighbor pairs reporting on the same household. The results of the kappa analysis therefore suggest the two preceding explanations for the differences between primary respondents and neighbor households are the most likely reasons for lower reported separation prevalences in neighbors’ households, compared to primary households. In other words, given the violation of two underlying assumptions of the method, the neighborhood method did not yield valid results in this setting.

### Limitations

This study is not without limitations. First, the survey had a very long recall period of 18 months. After discussion with local leadership, the M23 attacks were the only emergency event in the recent past that resonated with the majority of the study population. This may explain some of the discrepancies between primary households and neighbor households if primary respondents were more likely to suffer recall biases with regards to knowledge about their neighbors’ households, compared to knowledge about their own households. This potential recall bias pertains particularly to the composition of the neighbors’ households prior to the M23 attacks. In order to further explore the effects of length of recall period, future investigations should pilot the neighborhood method in a rapid-onset emergency setting with a shorter recall period.

Second, because child-headed households were excluded from primary households by design (all respondents had to be at least 18 years of age), unaccompanied children were systematically undercounted in primary households. Child-headed households were only included in the sub-sample of neighbor households. As a result, it is not appropriate to compare the prevalence of unaccompanied children in primary households to the prevalence of unaccompanied children in neighbors’ households. In fact, because of the systematic undercounting of unaccompanied children in primary households, but not in neighbors’ households, one might expect the prevalence of unaccompanied children to be higher in the neighbors’ households than in the primary households. That the prevalences were similar suggests that the prevalence in the neighbors’ households may be an undercount, consistent with the directionality of the other primary/neighbor prevalences compared. This bias could be partly addressed by widening the age criteria for interview eligibility to 15 years or older. Many well-established household surveys, such as the Demographic and Health Survey (DHS), already interview individuals 15 years and older as part of standard practice.

Finally, this pilot used a small sample size of neighbor pairs (*n* = 44) and a small sample size of households in camps (*n* = 104). The former limited the power of our reliability analysis and the latter limited our ability to draw meaningful conclusions about the appropriateness of using the neighborhood method in camp versus non-camp settings. In future investigations, the sample size for neighbor pairs and camps should be powered to explore these issues.

## Conclusion

By reducing sample size requirements, study time and costs, the neighborhood method holds potential to increase efficiency in data collection in emergencies. However, given the results of this analysis with regards to the comparability and reliability of the neighborhood method, we conclude that the neighborhood method is not a valid method for measuring separation in this setting. It is recommended that the neighborhood method be tested in the context of an acute-onset emergency with a shorter recall period and that the survey tool be revised to ask respondents about only one neighbor. It is hoped that, with these adaptations, the neighborhood method can save precious time and resources in humanitarian emergencies, without sacrificing data quality. Ultimately, the appropriateness of the neighborhood method for measuring separated children in emergencies hinges on demonstrating greater comparability of the main outcomes and improved reliability in other contexts. The neighborhood method cannot be recommended to measure separated children unless future pilots in settings more comparable to those for which this tool was developed (shorter recall period, acute-onset emergency) consistently provide valid results.

## Footnotes

1 The Child Protection Working Group (CPWG) is the global level forum for coordination and collaboration on child protection in humanitarian settings. The group brings together NGOs, UN agencies, academics and other partners under the shared objective of ensuring more predictable, accountable and effective child protection responses in emergencies (CPWG.net).

2 “Newborns” were defined as babies 18 months or younger, born after the M23 attack of interest.
